# Visceral Obesity Is a More Important Factor for Colorectal Adenomas than Skeletal Muscle or Body Fat

**DOI:** 10.3390/cancers14215256

**Published:** 2022-10-26

**Authors:** Ji Yeon Seo, Yoo Min Han, Su Jin Chung, Seon Hee Lim, Jung Ho Bae, Goh Eun Chung

**Affiliations:** Department of Internal Medicine and Healthcare Research Institute, Healthcare System Gangnam Center, Seoul National University Hospital, 39F Gangnam Finance Center 152, Teheran-ro, Gangnam-gu, Seoul 06236, Korea

**Keywords:** visceral fat, colorectal adenoma, bioelectrical impedance analyses

## Abstract

**Simple Summary:**

We aimed to determine which of the various body compositions best reflects the risk of colorectal adenoma. Five body indices, including skeletal muscle index, fat mass index, muscle-to-fat ratio, visceral fat index, and skeletal muscle mass-to-visceral fat area ratio, measured by bioelectrical impedance analysis, reflected the risk of adenoma and high-risk adenoma. Among the various body indices, visceral fat index was the most important index associated with the risk of colorectal adenoma. We suggest that more attention should be paid to visceral fat when predicting the risk of colorectal adenoma.

**Abstract:**

Objective: Although the incidence of colorectal adenomas increases with obesity, the role of various body compositions is still unknown. We aimed to determine which body composition best reflects the risk of colorectal adenomas. Methods: Patients aged 50–75 years who underwent colonoscopy and a bioelectrical impedance analysis (BIA) for a health check-up from 2017 to 2019 were retrospectively enrolled. The skeletal muscle index (SMI), fat mass index (FMI), and visceral fat index (VFI) were obtained using BIA. The muscle-to-fat ratio (MFR) and the skeletal muscle mass to visceral fat area ratio (SVR) were calculated. Results: Among 15,102 patients, 6605 (43.7%) had adenomas. SMI, FMI, MFR, VFI, and SVR were all associated with the risk of adenomas and high-risk adenomas in the age- and sex-adjusted model. When further adjusted for metabolic and lifestyle factors, VFI was the only factor significantly associated with the risk of colorectal adenomas and high-risk adenomas (adenomas: odds ratio (OR) 1.40, 95% confidence interval (CI) 1.22–1.61; high-risk adenomas: OR 1.47, 95% CI 1.16–1.87, for the highest quartile). Conclusion: Among the various body indices, VFI was the most important index associated with the development of colorectal adenoma. More attention should be paid to visceral fat when predicting the risk of colorectal adenomas.

## 1. Introduction

Colorectal cancer (CRC) is the third most common cancer and second leading cause of cancer-related deaths worldwide. In 2020, there were more than 1.9 million newly diagnosed cases of CRC and 935,000 deaths caused by CRC [1]. Most CRCs are derived from adenomas. Adenomas, with the accumulation of genetic alterations, progress to advanced adenomas and CRCs, the so-called adenoma–carcinoma sequence [2]. Therefore, the early detection and removal of colorectal adenomas is important to prevent CRC [3,4]. Colonoscopy is an effective tool for screening and removing precursor lesions of CRC. In fact, the introduction of screening colonoscopies effectively reduced the high incidence of CRC in the United States [5]. However, the excessive use of screening colonoscopies could be a socioeconomic burden. Therefore, it is important to select patients at high risk of CRC.

Several risk factors are known to increase the risk of CRC, including a westernized diet, decreased physical activity, heavy alcohol consumption, smoking, and processed meat [1,6]. Obesity is also a known risk factor for CRC, and its importance as a risk factor for various metabolic diseases and cancers are being emphasized [7]. Many studies have focused on the relationship between body mass index (BMI) or waist circumference (WC) and CRC or adenomas [8,9]. Several studies have shown a relationship between body fat mass [10,11], visceral fat area (VFA) [12], and CRC. VFA measured using computed tomography (CT) scans has been widely studied and shown to have a positive relationship with adenomas [13]. In a longitudinal study, increased VFA increased both incident and recurrent colorectal adenomas [14].

To measure VFA, CT, magnetic resonance imaging, and dual-energy X-ray absorptiometry are commonly used. Among them, CT is considered the gold standard for measuring abdominal VFA [15], however, CT has limitations in that it has a radiation hazard and is expensive. Recently, bioelectrical impedance analysis (BIA) has been widely used in health checkup centers and gyms in Korea and BIA is a simple, noninvasive, and inexpensive method to measure body composition [16]. Correlation analyses between BIA and CT images have been reported previously, and BIA can be a good alternative to CT scan [17,18].

On the other hand, low muscle mass or sarcopenia has also been reported as a risk factor for colorectal neoplasm [19,20]. Among these various body compositions, it is unknown which body composition best reflects the risk of colorectal neoplasm. The importance of body, muscle, and visceral fat has not yet been compared. Therefore, we investigated the relationship between various body indices and colonic neoplasia in an asymptomatic population.

## 2. Methods

### 2.1. Study Population

We conducted a retrospective cohort study. Patients aged 50–75 years who underwent colonoscopy at the Seoul National University Hospital (SNUH) Healthcare System Gangnam Center from June 2017 to December 2019 were retrospectively enrolled. Patients who underwent a BIA on the same day as colonoscopy were included in the study. Those with a history of inflammatory bowel disease (*n* = 70), a previous history of CRC or colorectal resection (*n* = 30), an inability to achieve cecal intubation, or inadequate bowel preparation (Boston Bowel Preparation Scale <6, *n* = 1058) were excluded. Finally, 15,102 participants were included in the analysis (Supplementary Figure S1). The study protocol was approved by the Institutional Review Board of SNUH (Institutional Review Board 2201-134-1294), and the requirement for informed consent was waived for the following reasons (i) This study involves no more than minimal risk to the subjects, (ii) The waiver or alteration will not adversely affect the rights and welfare of the subjects and (iii) The research could not practicably be carried out without the waiver or alteration. This study conformed to the ethical guidelines of the Declaration of Helsinki of the World Medical Association.

### 2.2. Clinical and Biochemical Evaluations

The study data consisted of medical information based on a self-administered questionnaire, as well as anthropometric and laboratory measurements. According to the self-reported questionnaire, the average alcohol intake was calculated and categorized into three groups: none, mild, and heavy (≥30 g of alcohol/day for men and ≥20 g of alcohol/day for women). Smoking status was classified as nonsmoker, ex-smoker, or current smoker. Family history of CRC was defined when first degree relatives were diagnosed as CRC. Hypertension was defined as systolic blood pressure ≥140 mmHg, diastolic blood pressure ≥ 90 mmHg, or a history of antihypertensive medications. Diabetes mellitus was defined as fasting glucose ≥126 mg/dL, glycated hemoglobin level ≥6.5%, or a history of glucose-lowering medications. Dyslipidemia was defined as total cholesterol ≥240 mg/dL, low-density lipoprotein ≥160 mg/dL, triglyceride ≥200 mg/dL, high-density lipoprotein <40 mg/dL, or a history of dyslipidemia medication. Patients fasted for 12 h prior to blood testing.

### 2.3. Anthropometric Measurements

Weight and height were measured by the medical staff using a digital scale. BMI was calculated as weight divided by height squared (kg/m^2^). WC was measured at the midpoint between the lower costal margin and the anterior superior iliac crest. An InBody 720 Body Composition Analyzer (InBody Co., Ltd., Seoul, Korea) was used to measure body composition. Participants were asked to stand with their feet touching and both hands grabbing the electrodes. When they stood with their legs slightly apart and both arms raised 45° away from the body, InBody performed multi-frequency measurements and checked the impedance of each segment, including the trunk and four limbs. The results provided appendicular skeletal muscle mass (ASM), body fat mass, and VFA. The principles and mechanisms have been previously reported in detail [17].

### 2.4. Definitions of Body Indices

ASM (kg) was calculated as the sum of the lean muscle masses of the four extremities. The skeletal muscle index (SMI (%)) was defined as ASM (kg)/body weight (kg) × 100 [19]. The fat muscle index (FMI (%)) was defined as measured body fat mass (kg)/body weight (kg) × 100. The muscle-to-fat ratio (MFR) was defined as ASM (kg)/body fat mass (kg) [21]. VFA (cm^2^) was estimated by the voltage flow from the flank to the umbilicus and back, which is not affected by subcutaneous fat and correlates with CT measurements [22]. VFA was adjusted by weight (VFA/weight, cm^2^/kg), which was defined as the visceral fat index (VFI) [23]. The skeletal muscle mass to VFA ratio (SVR) was calculated as ASM (kg) divided by VFA (cm^2^) [24]. Since there are no standardized cut-off values for these indices, all indices were divided into quartiles and analyzed.

### 2.5. Colonoscopic Examination

At our institution, colonoscopy is recommended in subjects over 45 years old according to the guidelines suggested by the U.S. Multi-Society Task Force on Colorectal Cancer [25]. If patients had family history of colorectal cancer, positive fecal hemoglobin or showed any symptoms, colonoscopy is done before age of 45. Follow up colonoscopy was performed according to the physician’s discretion based on the baseline endoscopic and histopathologic findings considering the average risk for colorectal cancer. Screening colonoscopy was defined as the first lifelong examination or performed after 10 years of negative colonoscopy. If a patient had a history of polyp and/or cancer, the purpose of colonoscopy has been classified as surveillance [26].

Colonoscopy was performed by 16 board-certified endoscopists who had performed at least 2000 procedures. A split-dose regimen of 2 L polyethylene glycol plus ascorbic acid (Coolprep; Taejoon Pharm, Seoul, Korea) was applied for bowel preparation, as previously described [27]. Polyps were removed or biopsied for second-stage removal, and the histology of all polyps was reviewed by board-certified pathologists of SNUH.

### 2.6. Outcomes

The primary outcome of this study was adenoma prevalence. The secondary outcome was the prevalence of high-risk adenomas and advanced neoplasms. High-risk adenomas were defined as ≥3 adenomas or advanced adenomas. An advanced adenoma is an adenoma with significant villous features (>25%), a size ≥1.0 cm, or high-grade dysplasia. Advanced neoplasm was defined as advanced adenomas or CRC. Advanced neoplasm was classified to the adenoma group.

### 2.7. Statistical Analyses

Data are expressed as numbers (percentages) for categorical variables and as mean ± standard deviation for continuous variables. The chi-square test was used to evaluate categorical variables, and the Wilcoxon rank-sum test was used to compare continuous variables. A logistic regression analysis was used to evaluate the risk of adenomas and high-risk adenomas. After the univariate analysis, variables with *p* < 0.10 were included in the multivariate analysis. Odds ratios (ORs) and 95% confidence intervals (CIs) were also calculated. Statistical analyses were performed using SAS version 9.4 (SAS Institute Inc., Cary, NC, USA). A *p* value of <0.05 was considered statistically significant.

## 3. Results

### 3.1. Baseline Characteristics of the Study Population

Th baseline characteristics of the study population are presented in Table 1. Among the 15,102 participants included in this study, 6605 (43.7%) had adenomas. The mean age was 59.1 ± 6.5 years, and the number of male patients was higher than that of female patients (57.3% vs. 42.7%). When the study patients were divided into adenoma and non-adenoma groups, higher rates of male sex, hypertension, diabetes, dyslipidemia, heavy drinkers, and current smokers were observed in the adenoma group (all *p* < 0.05). Both BMI and WC were significantly higher in the adenoma group, and all five body indices showed significant differences between the two groups (all *p* < 0.05). However, there was no difference in the purpose of colonoscopy or in the family history of CRC between the two groups. Baseline features according to the high-risk adenoma are shown in Supplementary Table S1.

### 3.2. Relationship between Various Body Indices and Adenomas

To analyze the importance of various body indices, each index was divided into four quartiles, and a logistic regression of the risk of adenomas, high-risk adenomas, and advanced neoplasms was performed after adjusting for age and sex (Table 2). The risks of adenomas, high-risk adenomas, and advanced neoplasms decreased with increasing SMI, MFR, and SVR. In contrast, the risks of adenomas, high-risk adenomas, and advanced neoplasms increased with increasing FMI and VFI. All *p* values were statistically significant, except for those of SMI and VFI, when assessing the risk of advanced neoplasms (*p* = 0.061 and *p* = 0.073, respectively).

### 3.3. Risk Factors for Adenomas and High-Risk Adenomas

A logistic regression was performed to identify factors associated with adenomas and high-risk adenomas (Table 3 and Table 4). In the univariate analysis, male sex, older age, hypertension, diabetes mellitus, dyslipidemia, alcohol intake, smoking, and all five body indices (SMI, FMI, MFR, VFI, and SVR) were significantly associated with adenomas (all *p* < 0.001). In the multivariate analysis, all demographic and metabolic factors were associated with adenomas, except for dyslipidemia (*p* = 0.569). Among the body indices, VFI was a significant risk factor for adenomas (OR, 1.40; 95% CI, 1.22–1.61 for Q4; *p* < 0.001). When VFI was excluded from the multivariate analysis, SVR was selected as a significant risk factor for adenomas (OR, 0.74; 95% CI, 0.64–0.85 for Q4; *p* < 0.001). Similarly, the multivariate analysis predicting the risk of high-risk adenomas showed that VFI was significant (OR, 1.47; 95% CI, 1.16–1.87 for Q4; *p* = 0.001), and SVR was significant (OR, 0.69; 95% CI, 0.53–0.88 for Q4; *p* = 0.003) when VFI was excluded.

### 3.4. Subgroup Analysis According to Body Mass Index

The risk of adenomas and high-risk adenomas associated with VFI was compared according to obesity (BMI ≥ 25 kg/m^2^) (Table 5). The risk of adenomas and high-risk adenomas was only significantly associated with VFI in the non-obese group (BMI < 25 kg/m^2^, *p* = 0.005 for adenomas and *p* = 0.006 for high-risk adenomas). The increased risk of adenoma and high-risk adenoma was significantly associated with VFI only in the < 70 years of age group but not in the ≥ 70 years of age group. When stratified by sex, these associations were significant in female and only in adenomas in male (Supplementary Table S2).

## 4. Discussion

Five body indices, namely, SMI, FMI, MFR, VFI, and SVR, measured by BIA, reflected the risk of adenomas and high-risk adenomas. Among them, VFI was the most significant predictor of adenomas and high-risk adenomas after adjusting for known risk factors for colorectal adenomas. Following VFI, SVR was the next most significant factor associated with adenomas and high-risk adenomas. The association between VFI and adenomas was significant in non-obese patients, suggesting the importance of body composition assessments in these participants.

The top two body indices selected in this study were VFI and SVR, which include VFA. Abdominal obesity was reported to be a stronger predictor of colorectal adenomas than BMI, which indicates general obesity [28,29]. In addition, visceral adipose tissue (VAT) is known to be associated with metabolic syndrome, regardless of BMI [30]. This indicates that VAT per se has a metabolic role. Insulin and insulin-like growth factor-1 (IGF-1) influence carcinogenesis by affecting cell proliferation and apoptosis. VAT promotes insulin resistance and hyperinsulinemia. High levels of insulin increase IGF-1 levels and result in a higher risk of colonic neoplasia [31]. VAT has been accepted as a metabolically active endocrine organ, and its importance is emphasized.

In contrast to VAT, the metabolic role of subcutaneous adipose tissue (SAT) is different. Several studies have suggested the favorable effects of SAT: SAT, as opposed to VAT, reduces all-cause mortality [32,33]. This protective effect can be interpreted by the fact that SAT is associated with a protective effect against insulin resistance [34]. SAT acts as a metabolic reservoir that can store lipids from circulation. Excessive fat beyond the capacity of SAT is stored in ectopic fat, the so-called VAT [35]. However, in terms of colonic adenomas, SAT was not a significant risk factor for colonic adenoma in previous studies [13,14,36]. Previous studies have reported that SAT correlates with fat mass measured using BIA. In our study, although we could not obtain SAT data, we regarded FMI as a marker of SAT [37,38]. In this study, the significant association between higher FMI and the higher risk of adenomas disappeared when adjusting for metabolic and lifestyle factors, suggesting that FMI is not a significant risk factor for adenomas. Further research is needed on the mechanism of action and the effects of FMI or SAT.

The relationship between sarcopenia or low skeletal muscle mass and the risk factor for colorectal adenomas has been previously reported [19,39]. Our results also show that an increase in SMI leads to a decreased risk of colorectal adenomas when adjusted for age and sex. Although the exact mechanism of the increase in adenomas with sarcopenia has not yet been verified, there are possible explanations. First, sarcopenia may be associated with other risk factors associated with colorectal adenomas, such as physical activity. Decreased physical activity might lead to decreased skeletal muscle, especially in old age [40]. Second, sarcopenia is related to abdominal obesity. Kim et al. reported that VFA was a predictor of the loss of skeletal muscle mass [41]. VAT itself is a risk factor for adenomas, and sarcopenia-associated visceral obesity increases blood pressure, triglycerides, and glucose, which are known risk factors for adenomas [42]. Lastly, similar to VAT, a decrease in skeletal muscle also affects insulin resistance, since muscle is a major site of glucose use [43].

Obesity is an obvious risk factor for colorectal adenomas. Therefore, we performed a subgroup analysis to determine whether there was a difference in the risk of adenomas according to the presence of obesity (BMI ≥ 25 kg/m^2^). As a result, VFI was only associated with adenomas or high-risk adenomas in non-obese patients (BMI < 25 kg/m^2^). Since obesity is already a known risk factor for colorectal adenomas and cancer [44,45], these findings could be interpreted as indicating that, in patients with obesity, the risk of obesity itself has a greater effect on adenomas than VFI. VFI is also associated with obesity; however, cytokines and inflammation relevant to pathologic obesity might outweigh the risk of visceral fat alone. Further research is needed to determine the exact mechanism of the relationship between VAT and obesity.

In our study, the adenoma detection rate (ADR) was greater than 40%, similar to that previously reported [26]. Several factors attributed the high ADR. We excluded participants with poor bowel preparation state (Boston Bowel Preparation Scale <6), and all the endoscopists in our institution were board-certified and had more than 7 years of colonoscopy experience and performed at least 1000 cases colonoscopies each year. Although a family history of CRC is related to an increased risk of metachronous colorectal adenoma [46], Kim et al. reported a differential association between a family history and colorectal adenoma according to the age groups that having first degree relatives with CRC was associated with an increased risk of metachronous colorectal neoplasm in the < 50 years of age group but not in the ≥50 years of age group [47]. Consistently, a family history of CRC was not significantly associated with adenoma in our study, and these results may be relevant to the age of our study population being over 50 years old.

To the best of our knowledge, this is the first study to compare the risk of colorectal neoplasia according to body composition. The effects of muscle, fat, and visceral fat were compared, and the importance of visceral fat was emphasized. However, this study has several limitations. First, this was a cross-sectional study, and it is difficult to determine cause and effect from these results. Second, since this study was performed at a single health check-up center, most participants were asymptomatic and solely interested in health, and there could be a selection bias. Third, only Koreans were included in this study; therefore, there are limitations in generalizing the results. Finally, although CT scan is regarded as the gold standard for assessing VFA, we could not present the correlation analysis results.

## 5. Conclusions

In conclusion, we demonstrated that, among the body indices measured by BIA, VFI was the most important index associated with the development of colorectal adenoma, especially in non-obese patients. Therefore, clinicians should pay more attention to visceral fat than to muscle or fat mass when interpreting BIA results when planning colonoscopies.

## Figures and Tables

**Table 1:** Baseline characteristics of study population according to adenoma.

	Total (*n* = 15,102)	No Adenoma (*n* = 8497)	Adenoma (*n* = 6605)	*p* Value
Age (years)	59.1 ± 6.5	58.3 ± 6.2	60.1 ± 6.6	<0.001
Male, *n* (%)	8651 (57.3)	4246 (50.0)	4405 (66.7)	<0.001
BMI (kg/m^2^)	23.6 ± 2.9	23.3 ± 2.9	24.0 ± 2.9	<0.001
WC (cm)	85.9 ± 8.3	84.9 ± 8.3	87.3 ± 8.2	<0.001
Hypertension, *n* (%)	5450/15,084 (36.1)	2721/8489 (32.1)	2729/6595 (41.4)	<0.001
Diabetes, *n* (%)	2134/15,084 (14.2)	990/8489 (11.7)	1144/6595 (17.4)	<0.001
Dyslipidemia, *n* (%)	8159/15,084 (54.1)	4434/8489 (52.2)	3725/6595 (56.5)	<0.001
Alcohol intake, *n* (%)	11,393	6310	5083	<0.001
—None	1308 (11.5)	833 (13.2)	475 (9.4)	
—Mild	8347 (73.3)	4668 (74.0)	3679 (72.4)	
—Heavy	1738 (15.3)	809 (12.8)	929 (18.3)	
Smoking, *n* (%)	13,862	7816	6046	<0.001
—Nonsmoker	7458 (53.8)	4693 (60.0)	2765 (45.7)	
—Ex-smoker	4366 (31.5)	2163 (27.7)	2203 (36.4)	
—Current smoker	2038 (14.7)	960 (12.3)	1078 (17.8)	
Purpose of colonoscopy, *n* (%)	14,979	8489	6595	0.240
—Screening	1168 (7.8)	639 (7.6)	529 (8.1)	
—Surveillance	13,811 (92.2)	7801 (92.4)	6010 (91.9)	
Family history of CRC, *n* (%)	1143/15,084 (7.6)	642/8489 (7.6)	501/6595 (7.6)	0.938
Skeletal muscle index	0.30 ± 0.04	0.30 ± 0.03	0.31 ± 0.03	<0.001
Fat mass index	0.27 ± 0.07	0.28 ± 0.07	0.27 ± 0.07	<0.001
Muscle/fat ratio	1.61 ± 0.63	1.59 ± 0.61	1.64 ± 0.64	<0.001
VFI (cm^2^/kg)	1.32 ± 0.34	1.33 ± 0.35	1.32 ± 0.34	0.002
SVR (kg/cm^2^)	0.34 ± 0.18	0.34 ± 0.18	0.34 ± 0.19	<0.001

Values are expressed as mean ± standard deviation or frequencies (percentages). BMI, body mass index; CRC, colorectal cancer; SVR, skeletal muscle mass/visceral fat area; VFI, visceral fat index; WC, waist circumference.

**Table 2:** The risk of adenomas, high-risk adenomas, and advanced neoplasms.

	SMI	FMI	MFR	VFI	SVR
Adenoma					
Q1	1 (reference)	1 (reference)	1 (reference)	1 (reference)	1 (reference)
Q2	0.85 (0.76–0.94)	1.02 (0.93–1.12)	0.87 (0.79–0.97)	1.10 (1.00–1.21)	0.87 (0.79–0.97)
Q3	0.82 (0.72–0.93)	1.15 (1.04–1.28)	0.76 (0.68–0.85)	1.16 (1.05–1.28)	0.80 (0.71–0.89)
Q4	0.74 (0.65–0.85)	1.35 (1.20–1.52)	0.76 (0.68–0.86)	1.32 (1.18–1.48)	0.76 (0.67–0.86)
*p* value	<0.001	<0.001	<0.001	<0.001	<0.001
High-risk adenoma					
Q1	1 (reference)	1 (reference)	1 (reference)	1 (reference)	1 (reference)
Q2	0.72 (0.59–0.89)	1.13 (0.96–1.32)	0.78 (0.65–0.94)	1.09 (0.93–1.28)	0.79 (0.66–0.96)
Q3	0.70 (0.56–0.88)	1.30 (1.09–1.54)	0.70 (0.58–0.86)	1.24 (1.05–1.47)	0.69 (0.57–0.84)
Q4	0.62 (0.49–0.79)	1.58 (1.29–1.93)	0.64 (0.52–0.79)	1.50 (1.22–1.82)	0.65 (0.53–0.80)
*p* value	0.001	<0.001	<0.001	0.001	<0.001
Advanced neoplasm					
Q1	1 (reference)	1 (reference)	1 (reference)	1 (reference)	1 (reference)
Q2	0.68 (0.49–0.94)	1.06 (0.82–1.38)	0.60 (0.45–0.81)	1.03 (0.78–1.34)	0.74 (0.55–0.99)
Q3	0.62 (0.43–0.89)	1.19 (0.89–1.58)	0.58 (0.43–0.80)	1.18 (0.89–1.55)	0.63 (0.46–0.86)
Q4	0.64 (0.44–0.93)	1.60 (1.16–2.22)	0.61 (0.44–0.84)	1.48 (1.08–2.04)	0.61 (0.44–0.85)
*p* value	0.061	0.025	0.002	0.073	0.017

Values are expressed as odds ratio (95% confidence intervals). Adjusted for age and sex. SMI: Q1 (SMI ≤ 0.28), Q2 (0.28 < SMI ≤ 0.31), Q3 (0.31 < SMI ≤ 0.33), Q4 (SMI > 0.33). FMI: Q1 (FMI ≤ 0.22), Q2 (0.22 < FMI ≤ 0.27), Q3 (0.27 < FMI ≤ 0.32), Q4 (FMI > 0.32). MFR: Q1 (MFR ≤ 1.17), Q2 (1.17 < MFR ≤ 1.52), Q3 (1.52 < MFR ≤ 1.93), Q4 (MFR > 1.93). VFI: Q1 (VFA1 ≤ 1.09), Q2 (1.09 < VFA1 ≤ 1.30), Q3 (1.30 < VFA1 ≤ 1.54), Q4 (VFA1 > 1.54). SVR: Q1 (SVR ≤ 0.24), Q2 (0.24 < SVR ≤ 0.31), Q3 (0.31 < SVR ≤ 0.40), Q4 (SVR > 0.40). FMI, fat mass index; MFR, muscle/fat ratio; Q, quartile; SMI, skeletal muscle index; SVR, skeletal muscle mass/visceral fat area; VFI, visceral fat index.

**Table 3:** The risk of adenomas according to various body indices.

	Univariate Model		Multivariate Model 1		Multivariate Model 2	
	OR (95% CI)	*p* Value	OR (95% CI)	*p* Value	OR (95% CI)	*p* Value
Sex (Male)	2.01(1.88–2.14)	<0.001	2.07 (1.82–2.35)	<0.001	2.07 (1.81–2.36)	<0.001
Age (years)		<0.001		<0.001		<0.001
50–69	1 (reference)		1 (reference)		1 (reference)	
70–75	1.66 (1.48–1.86)		1.61 (1.37–1.89)		1.62 (1.38–1.91)	
Hypertension	1.50 (1.40–1.60)	<0.001	1.16 (1.07–1.26)	<0.001	1.17 (1.07–1.27)	<0.001
Diabetes mellitus	1.59 (1.45–1.74)	<0.001	1.22 (1.09–1.37)	<0.001	1.23 (1.10–1.37)	<0.001
Dyslipidemia	1.19 (1.11–1.27)	<0.001	1.02 (0.95–1.11)	0.569	1.03 (0.95–1.11)	0.509
Alcohol intake		<0.001		0.002		0.002
—None	1 (reference)		1 (reference)		1 (reference)	
—Mild	1.38 (1.23–1.56)	<0.001	1.09 (0.96–1.25)	0.194	1.10 (0.96–1.25)	0.194
—Heavy	2.01 (1.74–2.33)	<0.001	1.31 (1.10–1.54)	0.002	1.31 (1.11–1.55)	0.002
Smoking		<0.001		0.003		0.002
—Nonsmoker	1 (reference)		1 (reference)		1 (reference)	
—Ex-smoker	1.73 (1.60–1.87)	<0.001	1.13 (1.01–1.26)	0.028	1.13 (1.02–1.26)	0.028
—Current smoker	1.91 (1.73–2.10)	<0.001	1.25 (1.10–1.42)	0.001	1.26 (1.10–1.43)	0.001
Purpose		0.239				
—Screening	1 (reference)					
—Surveillance	0.93 (0.83–1.05)					
FHx of CRC	1.01 (0.89–1.14)	0.938				
SMI		<0.001				
—Q1	1 (reference)					
—Q2	1.21 (1.11–1.32)	<0.001				
—Q3	1.56 (1.42–1.71)	<0.001				
—Q4	1.48 (1.35–1.63)	<0.001				
FMI		<0.001				
—Q1	1 (reference)					
—Q2	0.93 (0.85–1.02)	0.111				
—Q3	0.85 (0.78–0.94)	0.001				
—Q4	0.77 (0.70–0.85)	<0.001				
MFR		<0.001				
—Q1	1 (reference)					
—Q2	1.16 (1.05–1.27)	0.003				
—Q3	1.25 (1.14–1.37)	<0.001				
—Q4	1.36 (1.24–1.50)	<0.001				
VFI		<0.001		<0.001	N/A	N/A
—Q1	1 (reference)		1 (reference)			
—Q2	1.11 (1.02–1.22)	0.024	1.07 (0.97–1.19)	0.195		
—Q3	1.02 (0.92–1.12)	0.758	1.14 (1.02–1.28)	0.019		
—Q4	0.89 (0.81–0.97)	0.010	1.40 (1.22–1.61)	<0.001		
SVR		<0.001				<0.001
—Q1	1 (reference)				1 (reference)	
—Q2	1.17 (1.06–1.28)	0.002			0.83 (0.73–0.95)	0.195
—Q3	1.29 (1.17–1.41)	<0.001			0.76 (0.66–0.87)	0.019
—Q4	1.22 (1.11–1.34)	<0.001			0.74 (0.64–0.85)	<0.001

*p* < 0.10 in univariate analysis was included in multivariate analysis. Multivariate models 1 and 2 were adjusted for sex, age, hypertension, diabetes, dyslipidemia, alcohol intake, smoking, purpose of colonoscopy, and family history of colorectal cancer. Multivariate model 2 was analyzed excluding VFI. CI, confidence interval; CRC, colorectal cancer; FHx, family history; FMI, fat mass index; MFR, muscle/fat ratio; N/A, not available; OR, odds ratio; N/A, not available; Q, quartile; SMI, skeletal muscle index; SVR, skeletal muscle mass/visceral fat area; VFI, visceral fat index.

**Table 4:** The risk of high-risk adenomas according to various body indices.

	Univariate Model		Multivariate Model 1		Multivariate Model 2	
	OR (95% CI)	*p* Value	OR (95% CI)	*p* Value	OR (95% CI)	*p* Value
Sex (Male)	2.29 (2.02–2.59)	<0.001	2.29(1.79–2.91)	<0.001	2.31(1.80–2.96)	<0.001
Age (years)		<0.001		<0.001		<0.001
50–69	1 (reference)		1 (reference)		1 (reference)	
70–75	2.06 (1.76–2.42)		1.92 (1.54–2.40)		1.94 (1.56–2.42)	
Hypertension	1.55 (1.39–1.73)	<0.001	1.16 (1.01–1.33)	0.042	1.16 (1.01–1.33)	0.038
Diabetes mellitus	1.86 (1.63–2.13)	<0.001	1.41 (1.19–1.66)	0.042	1.41 (1.19–1.66)	0.038
Dyslipidemia	1.26 (1.13–1.41)	<0.001	1.10 (0.96–1.26)	0.185	1.10 (0.96–1.26)	0.172
Alcohol intake		<0.001		0.065		0.063
—None	1 (reference)		1 (reference)		1 (reference)	
—Mild	1.57 (1.24–1.99)	<0.001	1.23 (0.95–1.60)	0.125	1.23 (0.95–1.61)	0.120
—Heavy	2.15 (1.65–2.81)	<0.001	1.42 (1.05–1.92)	0.024	1.42 (1.05–1.93)	0.023
Smoking		<0.001		0.017		0.016
—Nonsmoker	1 (reference)		1 (reference)		1 (reference)	
—Ex-smoker	1.91 (1.67–2.17)	<0.001	1.23 (1.02–1.48)	0.031	1.23 (1.02–1.48)	0.030
—Current smoker	2.06 (1.76–2.42)	<0.001	1.35 (1.10–1.67)	0.005	1.35 (1.10–1.67)	0.005
Purpose		<0.001		<0.001		<0.001
—Screening	1 (reference)		1 (reference)		1 (reference)	
—Surveillance	0.55 (0.47–0.66)		0.46 (0.37–0.57)		0.46 (0.37–0.57)	
FHx of CRC	1.06 (0.87–1.30)	0.575				
SMI		<0.001				
—Q1	1 (reference)					
—Q2	1.24 (1.06–1.46)	0.009				
—Q3	1.59 (1.35–1.87)	<0.001				
—Q4	1.47 (1.25–1.74)	<0.001				
FMI		0.038				
—Q1	1 (reference)					
—Q2	1.03 (0.88–1.21)	0.688				
—Q3	0.97 (0.82–1.14)	0.668				
—Q4	0.82 (0.70–0.98)	0.025				
MFR		0.007				
—Q1	1 (reference)					
—Q2	1.16 (0.99–1.38)	0.074				
—Q3	1.29 (1.10–1.52)	0.002				
—Q4	1.28 (1.09–1.51)	0.003				
VFI		0.066		0.006	N/A	N/A
—Q1	1 (reference)		1 (reference)			
—Q2	1.13 (0.97–1.33)	0.120	1.09 (0.91–1.30)	0.350		
—Q3	1.13 (0.97–1.33)	0.124	1.27 (1.06–1.54)	0.011		
—Q4	0.95 (0.81–1.12)	0.547	1.47 (1.16–1.87)	0.001		
SVR		0.043				0.016
—Q1	1 (reference)				1 (reference)	
—Q2	1.20 (1.02–1.41)	0.031			0.84 (0.67–1.06)	0.148
—Q3	1.25 (1.07–1.47)	0.006			0.73 (0.58–0.93)	0.011
—Q4	1.14 (0.96–1.34)	0.132			0.69 (0.53–0.88)	0.003

*p* < 0.10 in univariate analysis was included in multivariate analysis. Multivariate models 1 and 2 were adjusted for sex, age, hypertension, diabetes, dyslipidemia, alcohol intake, smoking, purpose of colonoscopy, and family history of colorectal cancer. Multivariate model 2 was analyzed excluding VFI. CI, confidence interval; CRC, colorectal cancer; FHx, family history; FMI, fat mass index; MFR, muscle/fat ratio; N/A, not available; OR, odds ratio; Q, quartile; SMI, skeletal muscle index; SVR, skeletal muscle mass/visceral fat area; VFI, visceral fat index.

**Table 5:** Stratified analysis of the risks of adenomas and high-risk adenomas according to visceral fat index.

	Adenoma		High-Risk Adenoma	
BMI < 25 kg/m^2^	OR (95% CI)	*p* value	OR (95% CI)	*p* value
VFI		0.005		0.006
—Q1	1 (reference)		1 (reference)	
—Q2	1.10 (0.97–1.24)	0.127	1.11 (0.90–1.37)	0.332
—Q3	1.15 (0.99–1.32)	0.060	1.28 (1.00–1.64)	0.051
—Q4	1.37 (1.16–1.63)	<0.001	1.80 (1.29–2.50)	0.001
BMI ≥ 25 kg/m^2^	OR (95% CI)	*p* value	OR (95% CI)	*p* value
VFI		0.342		0.468
—Q1	1 (reference)		1 (reference)	
—Q2	0.97 (0.78–1.21)	0.816	1.09 (0.76–1.56)	0.649
—Q3	1.06 (0.85–1.33)	0.588	1.28 (0.90–1.83)	0.167
—Q4	1.21 (0.92–1.59)	0.177	1.14 (0.75–1.75)	0.538

Adjusted for sex, age, hypertension, diabetes, dyslipidemia, alcohol intake, smoking, purpose of colonoscopy and family history of CRC. BMI, body mass index (kg/m^2^); CI, confidence interval; CRC, colorectal cancer; OR, odds ratio; Q, quartile; VFI, visceral fat index.

## Data Availability

The data presented in this study are available on request from the corresponding author.

## Supplementary Materials

The following supporting information can be downloaded at: https://www.mdpi.com/article/10.3390/cancers14215256/s1, Figure S1. Flow chart of the study population; Table S1. Baseline characteristics of study population according to high-risk adenoma; Table S2. Stratified analysis for the risk of adenoma and high-risk adenoma.

## Abbreviations

ADR, adenoma detection rate; ASM: appendicular skeletal muscle mass; BIA, bioelectrical impedance analysis; BMI, body mass index; CI, confidence interval; CRC, colorectal cancer; CT, computed tomography; DXA, dual-energy X-ray absorptiometry; FMI, fat mass index; IGF-1, insulin-like growth factor-1; MFR, muscle-to-fat ratio; MRI, magnetic resonance imaging; OR, odds ratio; SAT, subcutaneous adipose tissue; SMI, skeletal muscle index; SNUH, Seoul National University Hospital; SVR, skeletal muscle mass to visceral fat area ratio; VAT, visceral adipose tissue; VFA, visceral fat area; VFI, visceral fat index; WC, waist circumference.
