# Supplementary material for: Visceral Obesity Is a More Important Factor for Colorectal Adenomas than Skeletal Muscle or Body Fat
Source: Cancers (Basel). 2022 Oct 26;14(21):5256. doi: 10.3390/cancers14215256 (PMC9653975; doi:10.3390/cancers14215256)
Supplement: Supplementary file 1 [file cancers-14-05256-s001.zip › cancers-1974155-supplementary.pdf]

cancers MDPI

Table S1. Baseline characteristics of study population according to high-risk adenoma.

	Total (n=15,102)	No high-risk adenoma (n=13,693)	High-risk adenoma (n=1,409)	*P* value
Age (years)	59.1 ± 6.5	58.9 ± 6.4	61.2 ± 6.7	<0.001
Male, n (%)	8,651 (57.3)	7,607 (55.6)	1,044 (74.1)	<0.001
BMI (kg/m^2)	23.6 ± 2.9	23.5 ± 2.9	24.3 ± 2.9	<0.001
WC (cm)	85.9 ± 8.3	85.6 ± 8.3	88.6 ± 7.9	<0.001
Hypertension, n (%)	5,450/15,084 (36.1)	4,807/13,676 (35.2)	643/1,408 (45.7)	<0.001
Diabetes, n (%)	2,134/15,084 (14.2)	1,821/13,676 (13.3)	313/1,408 (22.2)	<0.001
Dyslipidemia, n (%)	8,159/15,084 (54.1)	7,324/13,676 (53.6)	835/1,408 (59.3)	<0.001
Alcohol intake, n (%)	11,393	10,311	1,082	<0.001
- None	1308 (11.5)	1,522 (14.8)	216 (20.0)	
- Mild	8347 (73.3)	7,562 (73.3)	785 (72.6)	
- Heavy	1738 (15.3)	1,227 (11.9)	81 (7.5)	
Smoking, n (%)	13,862	12,603	1,259	<0.001
- Non smoker	7,458 (53.8)	6,970 (55.3)	488 (38.8)	
- Ex smoker	4,366 (31.5)	3,852 (30.6)	514 (40.8)	
- Current smoker	2,038 (14.7)	1,781 (14.1)	257 (20.4)	
Purpose of colonoscopy, n (%)	14,979	13,586	1,393	<0.001
- Screening	1,168 (7.8)	994 (7.3)	174 (12.5)	
- Surveillance	13,811 (92.2)	12,592 (92.7)	1,219 (87.5)	
Family history of CRC, n (%)	1,143/15,084 (7.6)	1,031/13,676 (7.5)	112/1,408 (8.0)	0.575
Skeletal muscle index	0.30 ± 0.04	0.30 ± 0.04	0.31 ± 0.03	<0.001
Fat mass index	0.27 ± 0.07	0.27 ± 0.07	0.27 ± 0.06	0.037
Muscle fat ratio	1.61 ± 0.63	1.61 ± 0.63	1.64 ± 0.58	0.008
VFI (cm^2/kg)	1.32 ± 0.34	1.32 ± 0.34	1.32 ± 0.33	0.755
SVR (kg/cm^2)	0.34 ± 0.18	0.34 ± 0.19	0.34 ± 0.13	0.185

Values are expressed as mean ± standard deviation or frequencies (percentages). BMI, body mass index; SVR, skeletal muscle mass/visceral fat area; CRC, colorectal cancer; VFI, visceral fat index; WC, waist circumference.

Table S2. Stratified analysis for the risk of adenoma and high-risk adenoma.

	Adenoma		High risk adenoma	
VFI	OR (95% CI)	*P* value	OR (95% CI)	*P* value
50≤Age<70		<0.001		0.009
- Q1	1 (reference)		1 (reference)	
- Q2	1.05 (0.95,1.17)	0.356	1.01 (0.84,1.21)	0.952
- Q3	1.11 (0.99,1.25)	0.067	1.23 (1.02,1.50)	0.034
- Q4	1.39 (1.20,1.60)	<0.001	1.44 (1.12,1.85)	0.005
Age≥70		0.089		0.014
- Q1	1 (reference)		1 (reference)	
- Q2	1.63 (0.97,2.75)	0.064	4.61 (1.84,11.56)	0.901
- Q3	1.85 (1.12,3.05)	0.017	3.62 (1.45,9.02)	0.199
- Q4	1.90 (1.07,3.37)	0.029	3.51 (1.30,9.51)	0.042
Male		0.019		0.084
- Q1	1 (reference)		1 (reference)	
- Q2	1.09 (0.98,1.22)	0.123	1.11 (0.92,1.33)	0.270
- Q3	1.18 (1.03,1.34)	0.013	1.28 (1.05,1.56)	0.014
- Q4	1.31 (1.07,1.62)	0.010	1.27 (0.94,1.72)	0.124
Female		0.002		0.035
- Q1	1 (reference)		1 (reference)	
- Q2	0.96 (0.72,1.28)	0.766	0.95 (0.43,2.09)	0.901
- Q3	1.04 (0.80,1.34)	0.784	1.54 (0.80,2.97)	0.199
- Q4	1.34 (1.05,1.71)	0.018	1.92 (1.02,3.59)	0.042

CI, confidence interval; OR, odds ratio; Q, quartile; VFI, visceral fat index. Adjusted for sex, age, hypertension, diabetes, dyslipidemia, alcohol intake, smoking, purpose of colonoscopy and family history of CRC.

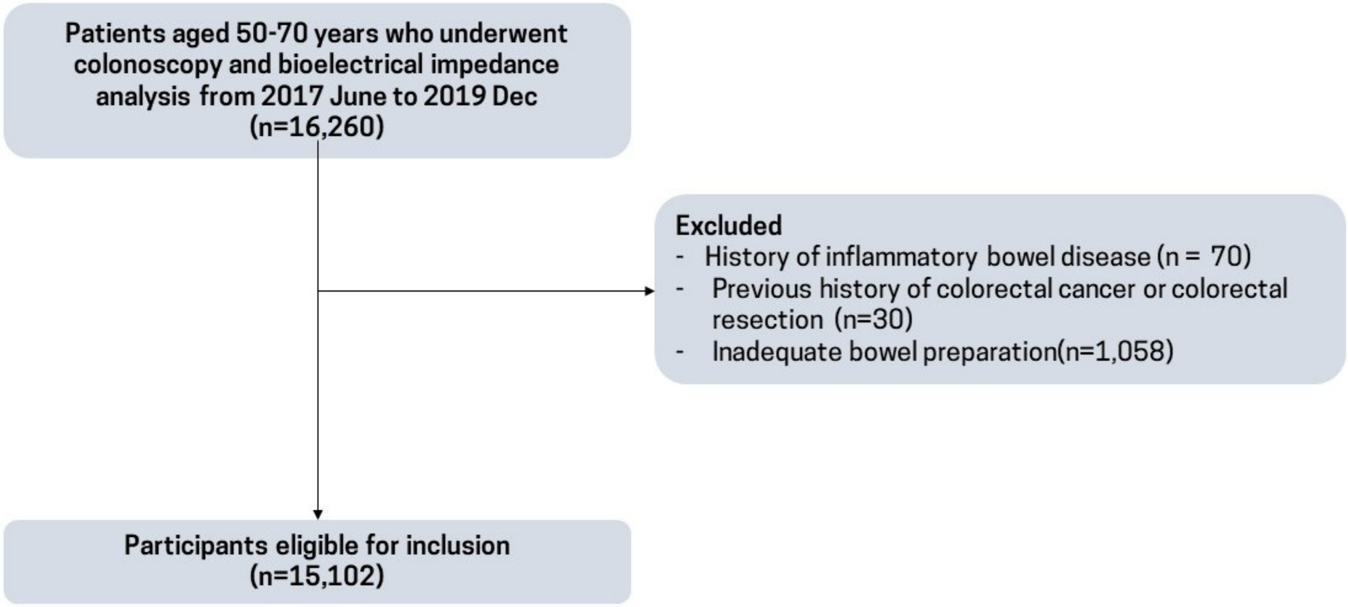

Figure S1. Flow chart of the study population.
